# Supplementary material for: Heavy Metals in Groundwater of Southern Italy: Occurrence and Potential Adverse Effects on the Environment and Human Health
Source: Int J Environ Res Public Health. 2023 Jan 17;20(3):1693. doi: 10.3390/ijerph20031693 (PMC9914834; doi:10.3390/ijerph20031693)
Supplement: Supplementary file 1 [file ijerph-20-01693-s001.zip › ijerph-2155997-supplementary.pdf]

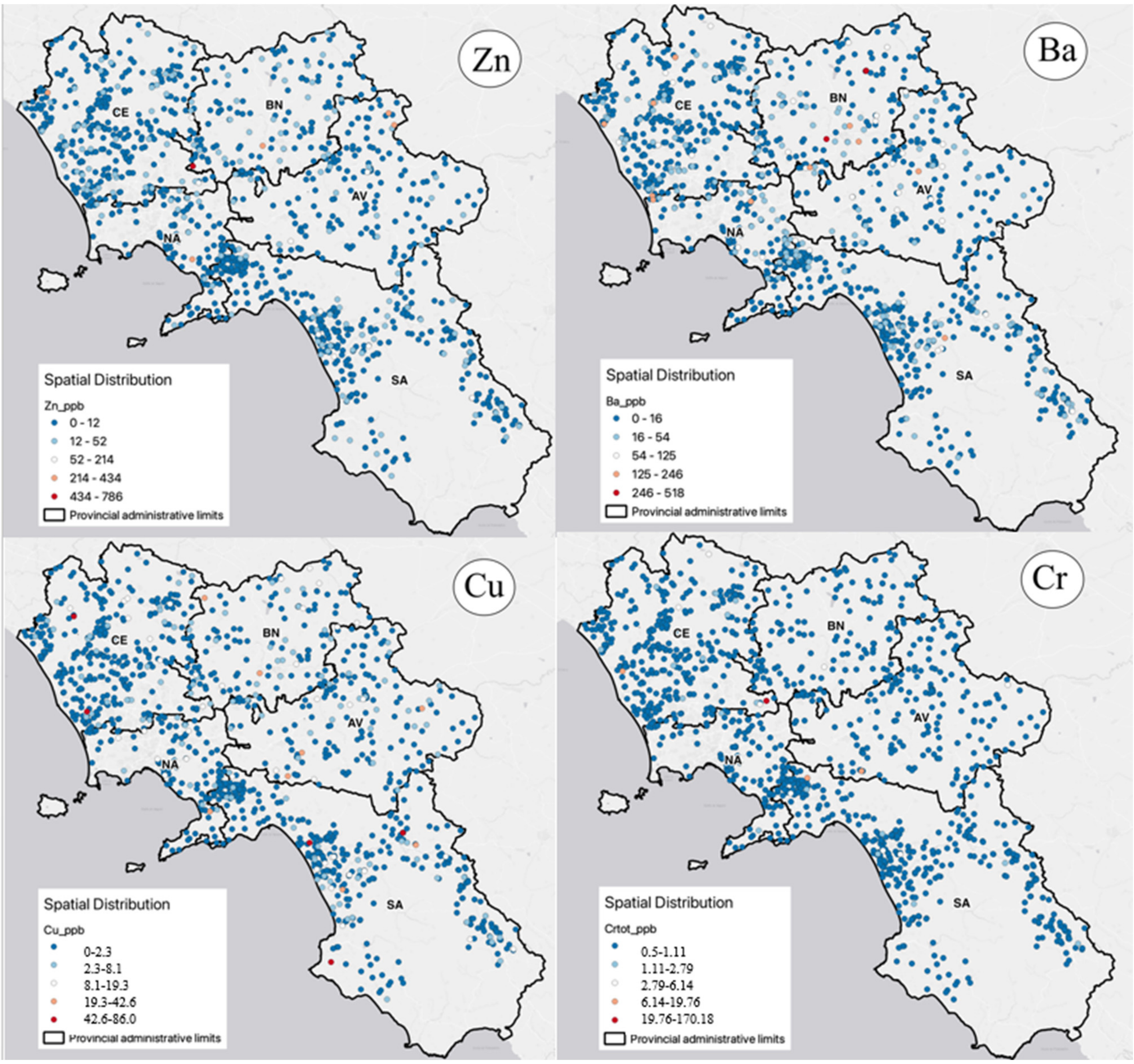

Figure S1. Heavy metal's distribution in Campania Plain (Zn, Ba, Cu, Cr)

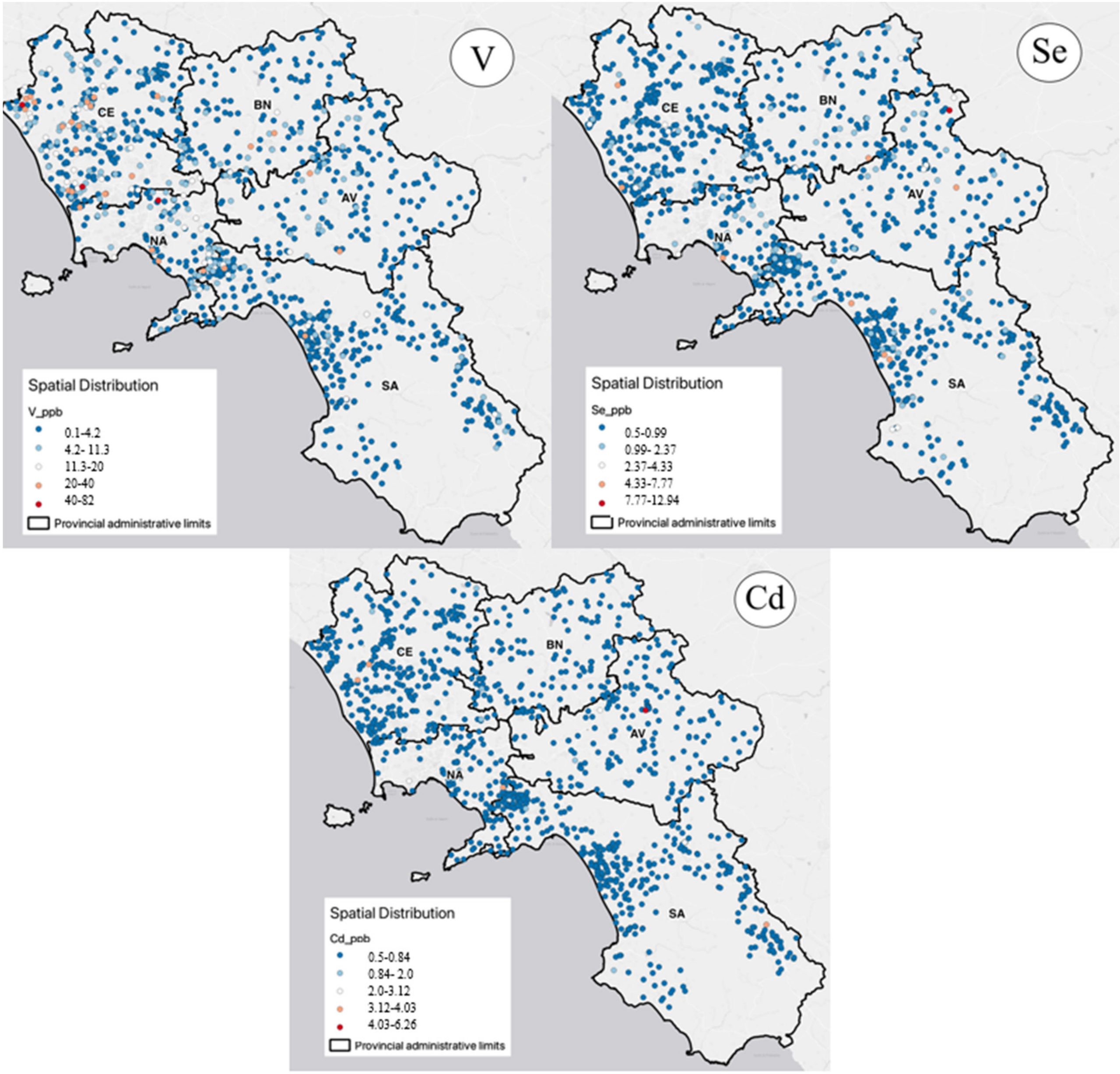

Figure S2. Heavy metal's distribution in Campania Plain (V, Se, Cd)

Table S1. Minimum, Maximum, Mean and Standard Deviation of the chemical-physical parameters (pH, Conductivity (EC) and Temperature (C°)) of the groundwater samples in the study area of the Campania Plain. The data analysed was grouped by province (Naples, Caserta, Salerno, Avellino and Benevento).

	**Minimum**	**Maximum**	**Mean**	**Std. Dev.**
**NAPLES**				
pH	6.1	8.7	7.1	1.12
Conductivity (µS/cm)	405	3650	1050	420
Temperature (C°)	8.1	18.2	12.2	6.20
**CASERTA**				
pH	7.5	9.1	8.1	0.90
Conductivity (µS/cm)	503	3008	1220	350
Temperature (C°)	6.2	15.0	9.2	7.0
**SALERNO**				
pH	6.5	8.0	7.5	1.10
Conductivity (µS/cm)	396	2269	1056	400
Temperature (C°)	7.3	16.0	10.0	7.5
**AVELLINO**				
pH	6.5	10.2	8.4	0.85
Conductivity (µS/cm)	480	2893	1032	390
Temperature (C°)	5.2	12.1	9.1	6.5
**BENEVENTO**				
pH	7.3	9.0	8.0	0.92
Conductivity (µS/cm)	496	2860	1240	460
Temperature (C°)	6.1	11.5	8.5	6.0
